# Supplementary material for: A high-throughput conditioned-media-based screening system identifies inhibitors of aggregation induced by iPSC-secreted amyloid β
Source: Nat Commun. 2026 Mar 24;17:4373. doi: 10.1038/s41467-026-71078-8 (PMC13179371; doi:10.1038/s41467-026-71078-8)
Supplement: Supplementary file 1 — Supplementary Information [file 41467_2026_71078_MOESM1_ESM.pdf]

Supplementary information

A high-throughput conditioned-media-based screening system identifies inhibitors of aggregation induced by iPSC-secreted amyloid β

Masahiro Kuragano[1], Naoki Nishishita[1,2#], Koki Araya[1], Akira Kobayashi[2], Taro Q.P. Noguchi[3], Kenichi Watanabe[4], Shinya Watanabe[1], Stefan Baar[1], Koji Uwai[1], and Kiyotaka Tokuraku[1#]

[1]Graduate School of Engineering, Muroran Institute of Technology, Hokkaido 050-8585, Japan
[2]Regenerative Medicine and Cell Therapy Laboratories, KANEKA CORPORATION, Kobe, 650-0047, Japan
[3]Department of Chemical Science and Engineering, National Institute of Technology, Miyakonojo College, Miyakonojo 885-8567, Japan
[4]Department of Veterinary Medicine, Research Center of Global Aeromedicine, Obihiro University of Agriculture and Veterinary Medicine, Obihiro 080-8555, Japan

[#]Contributed equally.
Corresponding author: Kiyotaka Tokuraku
E-mail: tokuraku@mmm.muroran-it.ac.jp
Corresponding author: Naoki Nishishita
E-mail: Naoki.Nishishita@kaneka.co.jp

Keywords: Alzheimer's disease, Induced pluripotent stem cells, Amyloid β, Tau, α-synuclein, Amyloid aggregation inhibitor, Quantum dot

Supplementary figures

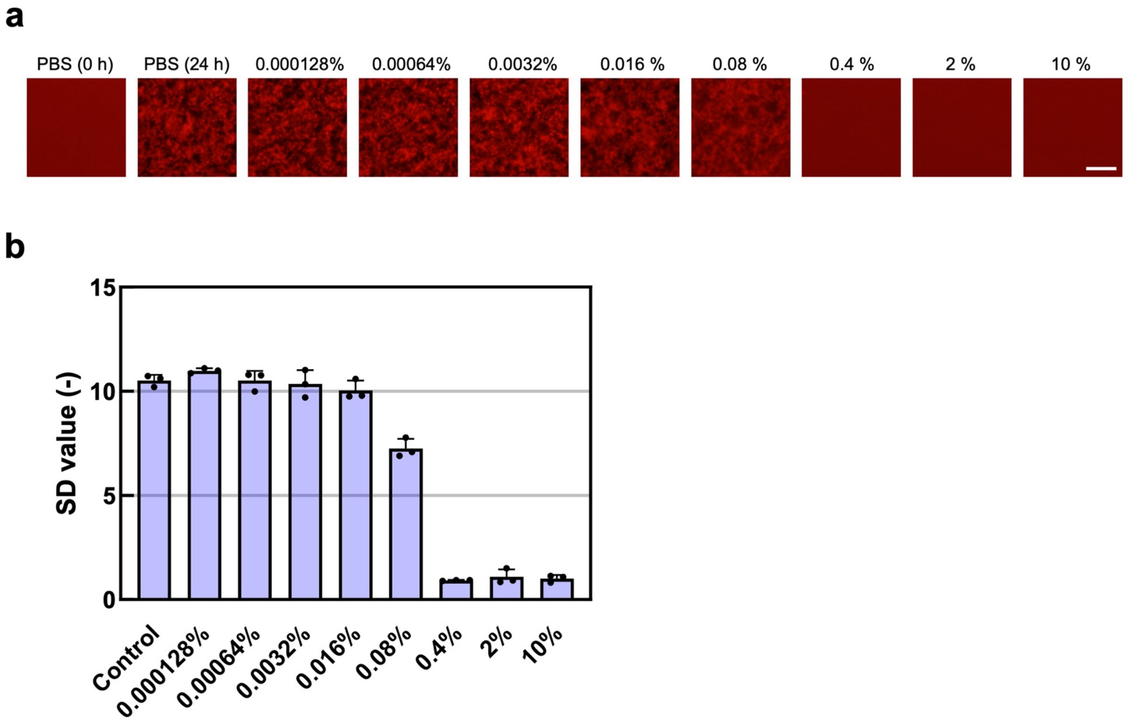

Supplementary Fig. 1. Effects of B-27 solution on Aβ aggregation. **a**, 2D imaging of Aβ aggregates within PBS supplemented with B-27 solution. Note that the formation of aggregates was strongly inhibited by the addition of 0.4–10% B-27 solution. Bar = 100 μm. **b**, Quantification of SD value in each condition. SD values were determined by ImageJ software using the 2D image. Error bars represent ± SDs of the mean values from fluorescence intensities (n = 3 separate experiments).

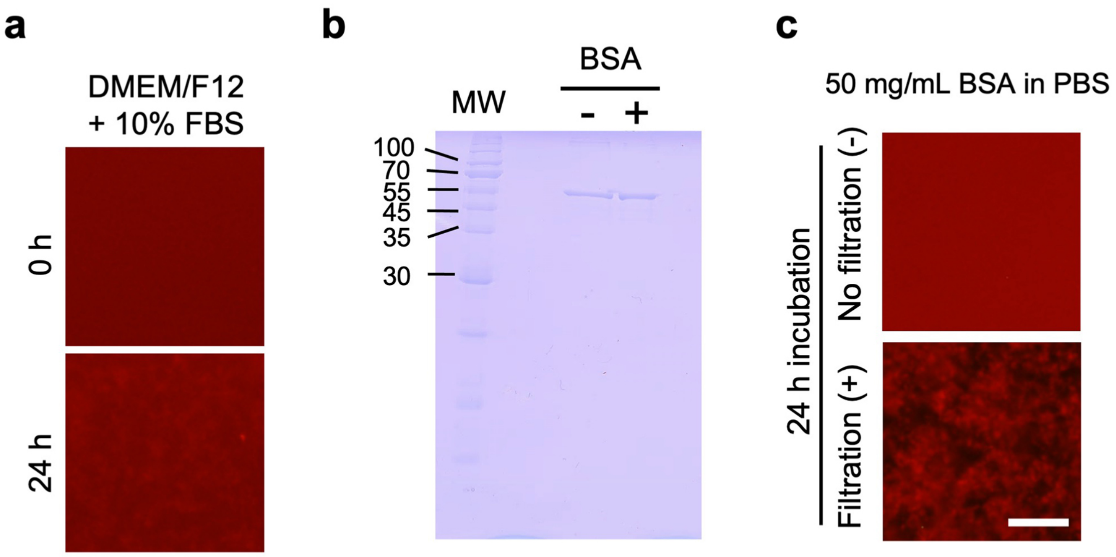

Supplementary Fig. 2. Effects of proteins on Aβ aggregation. a, 2D imaging of Aβ aggregates within DMEM/F12 medium supplemented with 10% FBS. Note that aggregates formed slightly in the condition for cell culture. **b**, Representative SDS-PAGE gel of 50 mg/mL BSA/PBS with (+) or without (-) filtration using a 50 kDa Amicon Ultra filter unit. Unfiltered 50 mg/mL BSA sample was diluted 100-fold and applied to the gel. **c**, 2D imaging of Aβ aggregates within 50 mg/mL BSA/PBS with or without filtration using a 50 kDa Amicon Ultra filter unit. Bar = 100 μm.

These results showed that BSA was reduced to 1/100th of its initial amount and was removed by filtration using the 50 kDa filter unit. Due to the removal of albumin, which has Aβ aggregation inhibitory activity, Aβ aggregates were observed in the filtered 50 mg/mL BSA solution, but not in the unfiltered BSA solution.

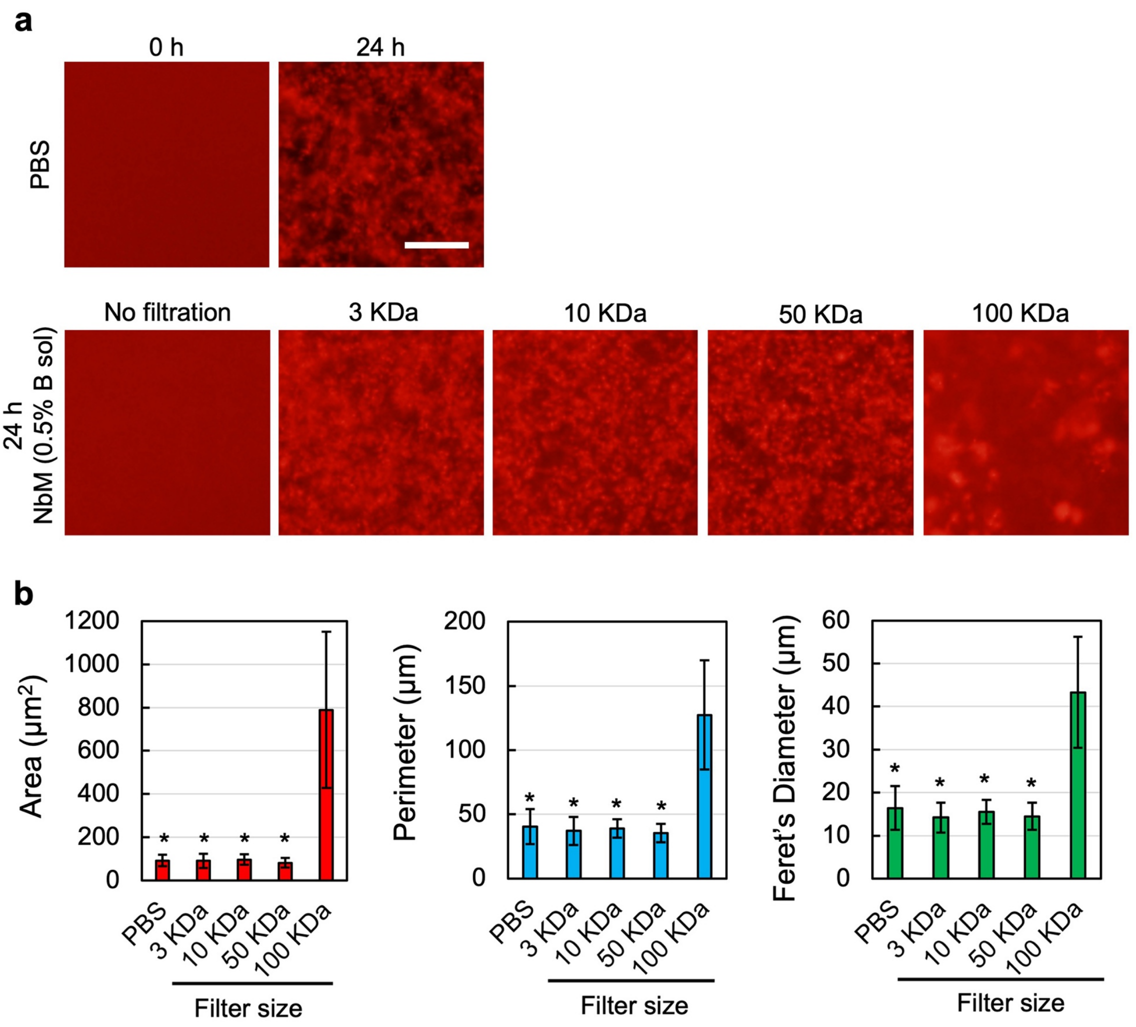

Supplementary Fig. 3. Aβ aggregation in the albumin-filtered medium. a, 2D imaging of Aβ aggregates within Neurobasal® Medium (NbM) with filtration using 3, 10, 50, and 100 kDa Amicon Ultra filter units. Note that abnormal aggregates formed in the condition using the 100 kDa Amicon Ultra filter unit. Aβ aggregates in PBS, as a positive control, were observed. All images were captured using a conventional fluorescence microscope. Bar = 100 μm. **b**, Quantification of Aβ aggregate size in each condition. Area (μm^2), perimeter (μm), and Feret's diameter (μm) were determined by ImageJ software using the 2D image. Error bars represent ± SDs of the mean values from fluorescence intensities ($n = 3$ separate experiments, $P < 0.05$, Kruskal-Wallis test).

Aβ aggregates between 3 and 50 kDa formed normally and displayed unmodified morphology in medium that was filtered by Amicon Ultra. However, when medium containing proteins greater than 100 kDa was filtered, normal aggregation was inhibited by the effects of albumin, even if abnormal aggregates with higher area, perimeter, and Feret's diameter emerged. In the latter

condition, serum albumin does not appear to have been eliminated from the medium. Therefore, Amicon Ultra that separated proteins greater than 50 kDa was most appropriate for our study.

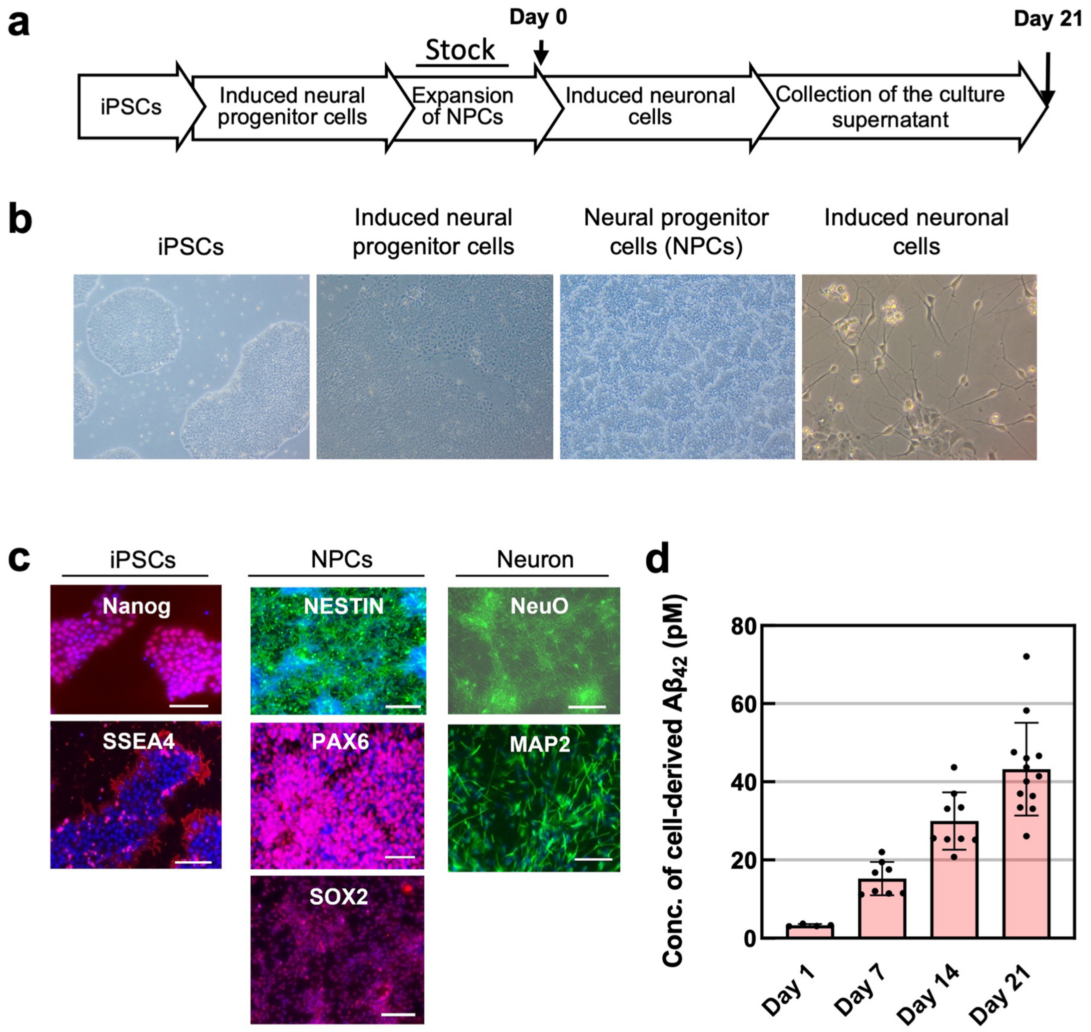

Supplementary Fig. 4. Establishment of AD-iNeuron. a, Scheme of neural cell induction from AD-iPS cells via neural progenitor cells. **b**, Representative cell images of the process of inducing iPS cells into neurons. **c**, Immuno-staining of iPSCs, NPCs and neurons, using several antibodies: anti-Nanog antibody (dilution ×250, host: rabbit, ab109250), anti-SSEA4 (dilution ×500, host: mouse, #MA1-021), anti-NESTIN (dilution ×150, host: mouse, #A24345), rabbit anti-PAX6 (dilution ×150, host: rabbit, #A24340), anti-SOX2 (dilution ×150, host: rabbit, #ab137358), anti-MAP2 (dilution ×250, host: mouse, #ab254144). Bar = 100 µm. **d**, Quantification of extracellular Aβ_{42} concentration in the supernatant after filtration of AD-iNeuron. Concentrations were assessed at 21-day differentiation culture periods. Error bars represent ± SDs of the mean values from n > 4 separate experiments.

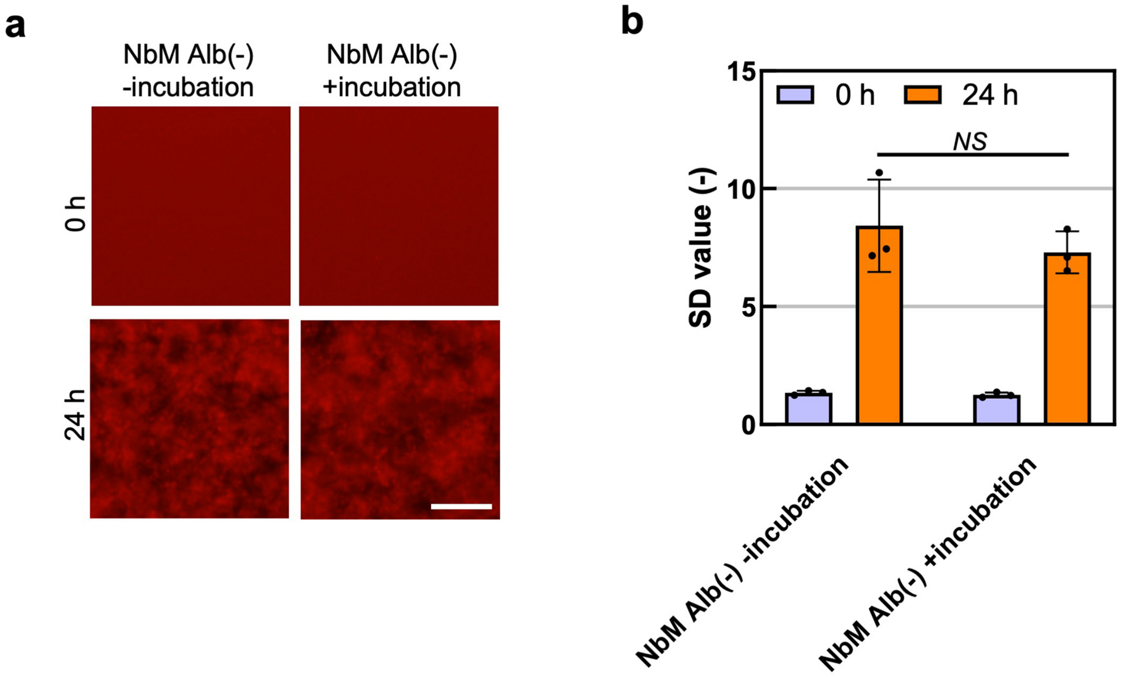

Supplementary Fig. 5. Effects of 37°C incubation on Aβ aggregation using NbM Alb(-). a, 2D imaging of Aβ aggregates in NbM Alb(-) with or without 37°C incubation overnight. Note that incubation without AD-iNeuron did not affect the morphology of Aβ aggregates. Bar = 100 μm. **b**, Quantification of SD value in each condition. SD values were determined by ImageJ software using the 2D image. Error bars represent ± SDs of the mean values from fluorescence intensities (n = 3 separate experiments, $p < 0.05$, Student's t-test).

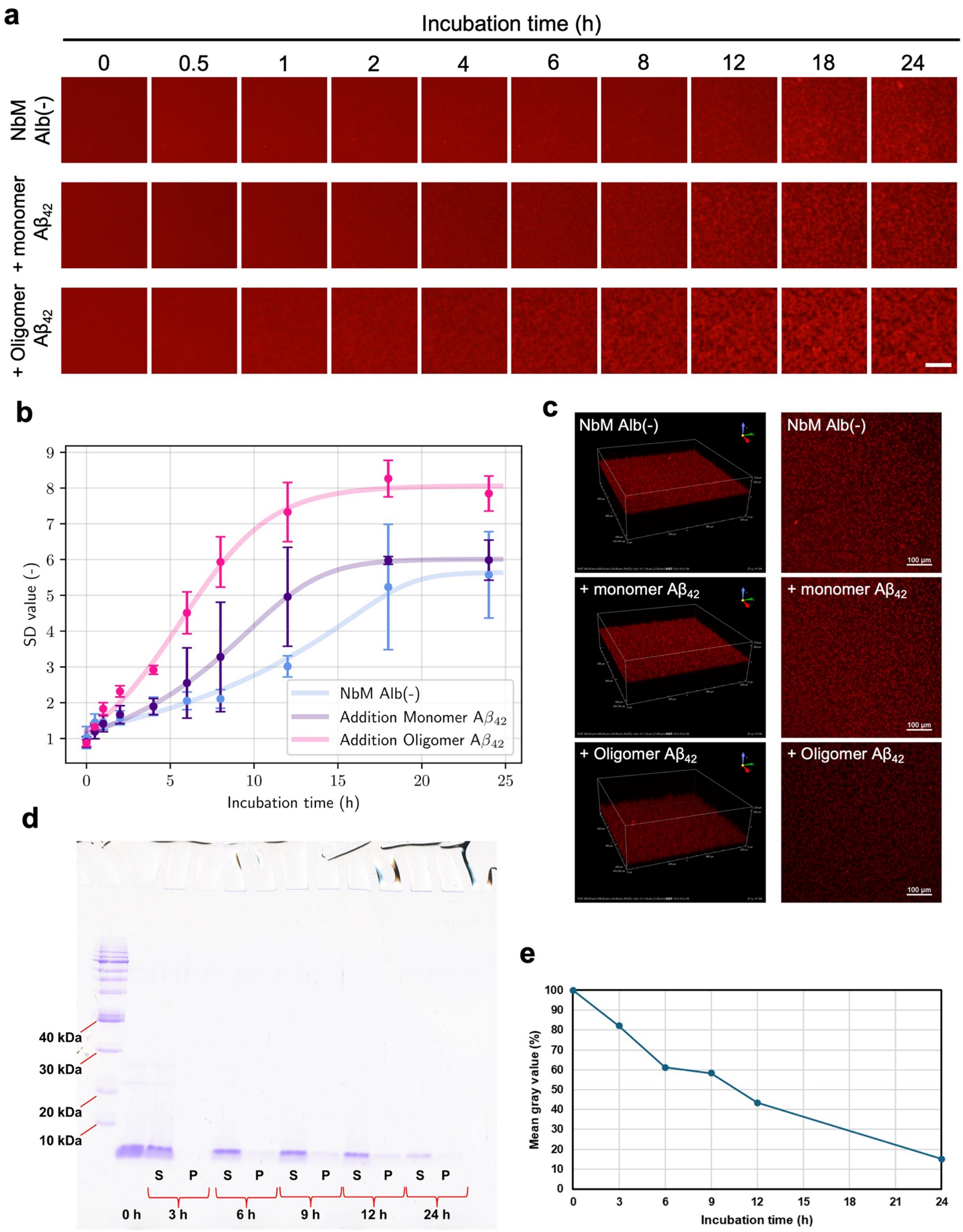

Supplementary Fig. 6. Acceleration of Aβ aggregation by the addition of Aβ monomer and oligomer. Oligomer $A\beta_{42}$ were prepared according to the report by Chau and Kim [Chau, E. & Kim, J.R., α-synuclein-assisted oligomerization of β-amyloid (1-42). Arch Biochem Biophys 717, 109120 (2022)]. **a**, Real-time imaging of Aβ aggregation processes in indicated conditions. 25 pM monomer $A\beta_{42}$ or 25 pM oligomer $A\beta_{42}$ were incubated in NbM Alb(-). All images were

captured using a conventional fluorescence microscope. Bar = 100 μm. **b**, Increase of SD values in each condition. SD values were determined by ImageJ software using the 2D images of Aβ. Error bars represent ± SDs of the mean values from fluorescence intensities (n = 3 separate experiments). **c**, 3D reconstruction images and slice images of Aβ aggregates in indicated conditions at 24 h of panel a. All images were captured using a confocal laser microscope. Scale bar = 100 μm. **d** and **e** show the results of monitoring the oligomerization process of Aβ monomers by SDS-PAGE. d, According to the method of Chau and Kim, the Aβ samples incubated for various time periods to oligomerize were centrifuged at 16,900 ×g for 5 min at 4°C, and the supernatant (S) and precipitate (P) were subjected to SDS-PAGE and stained by Coomassie Brilliant Blue (CBB). **e**, The band at the position of Aβ monomers (4.5 kDa) in the supernatant was surrounded by an area of the same size, and its Mean Gray value was quantified using ImageJ software (NIH). The decrease in Aβ monomers suggests that Aβ aggregates into oligomers in a time-dependent manner, although it should be noted that the oligomers exist in various sizes and are present in small amounts, making them undetectable by CBB staining. In these experiments (a, b, and c), the supernatant of a sample 12 h after oligomerization, in which just over half of the Aβ monomers had oligomerized, was added as an oligomer solution to the NbM Alb(-) sample. Therefore, it is estimated that the amount of oligomers contained is approximately 15 pM.

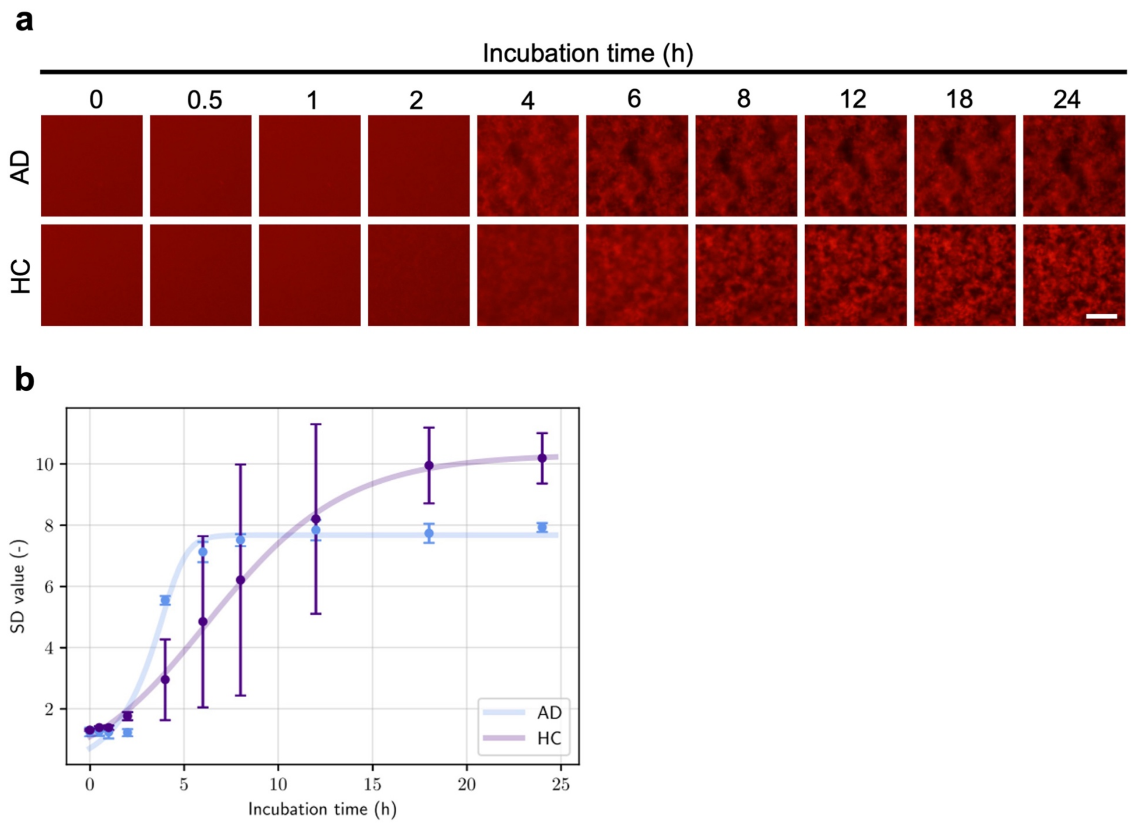

Supplementary Fig. 7. Comparison of Aβ aggregation rate between AD iNeuron-derived NbM Sup Alb(-) and HC iNeuron-derived NbM Sup Alb(-). a, Real-time imaging of Aβ aggregation processes in indicated conditions. 25 µM $A\beta_{42}$ and 25 nM $QDA\beta_{40}$ were incubated in AD or HC iNeuron-derived NbM Sup Alb(-). All images were captured using a conventional fluorescence microscope. Bar = 100 µm. **b**, Increase of SD values in each condition. SD values were determined by ImageJ software using the 2D images of Aβ. Error bars represent ± SDs of the mean values from fluorescence intensities (n = 3 separate experiments).

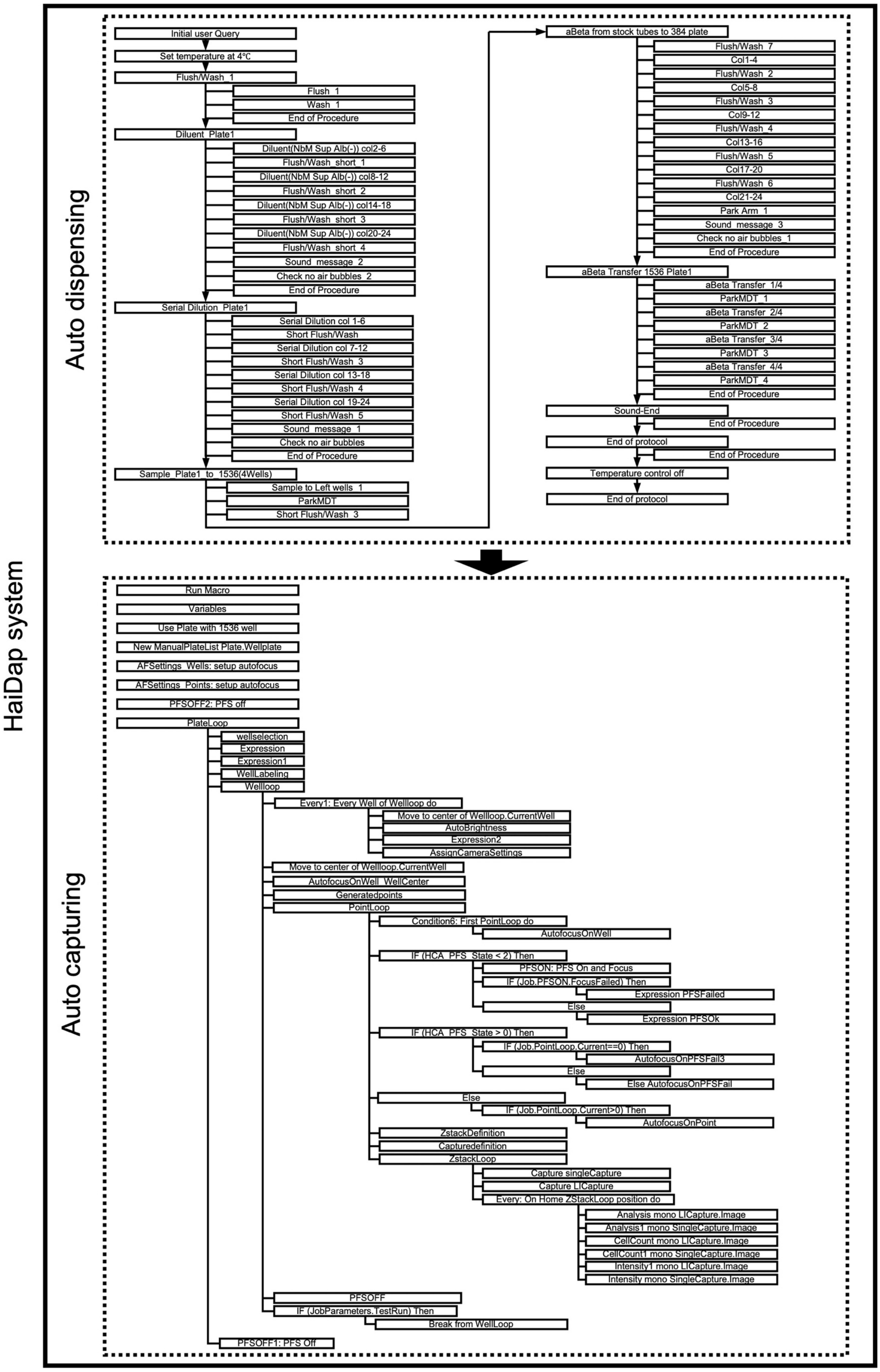

Supplementary Fig. 8. Scheme of the HaiDap system. The HaiDap system is mainly divided into two parts, the auto-dispensing part and the auto-capturing part. In the auto-dispensing part,

sample preparation and mixing with Aβ were carried out using Automated Workstation JANUS G3 (Perkin Elmer). In the auto-capturing part, the 1536-well plate was observed by an inverted fluorescence microscope system (ECLIPSE Ti-E, Nikon) equipped with a color CMOS camera (DS-Ri2, Nikon). QDs were imaged using a 4× objective lens and a TRITC filter set (TRITC-A-Basic-NTE, Semrock).

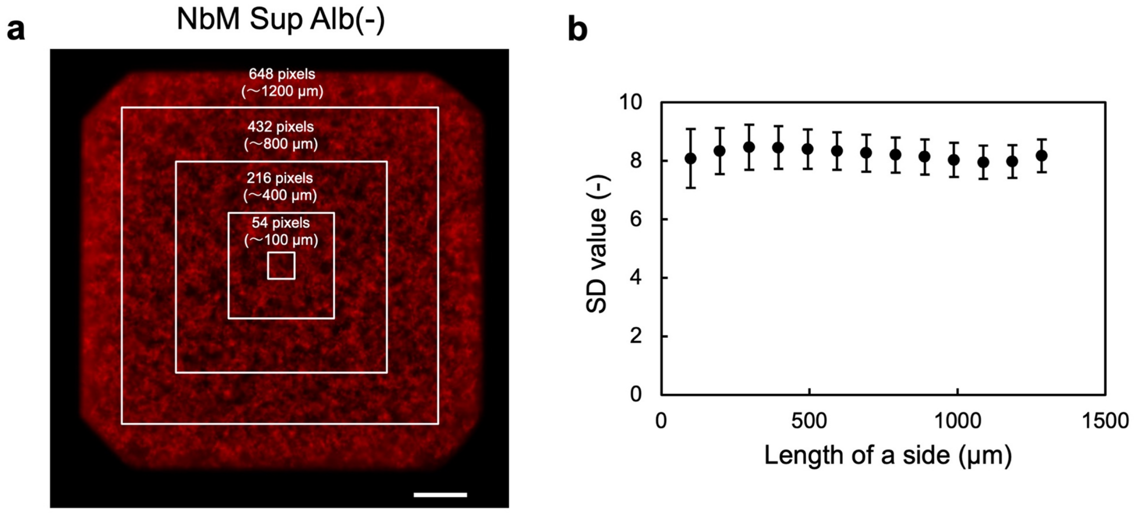

Supplementary Fig. 9. Relationship between size of the imaging area and SD value. a, 25 μM Aβ and 25 nM QDAβ were incubated in a 1536-well plate for 24 h at 37°C, and wells were observed by fluorescence microscopy. Fluorescence microscopic images of typical Aβ aggregate in NbM Sup Alb(-). **b**, Average SD values determined from various imaging areas in the central well region of six wells. Since 54 pixels is approximately 100 μm in this imaging system, we gradually increased the imaging area based on 54 pixels. Bar = 100 μm. All images were captured using a conventional fluorescence microscope.

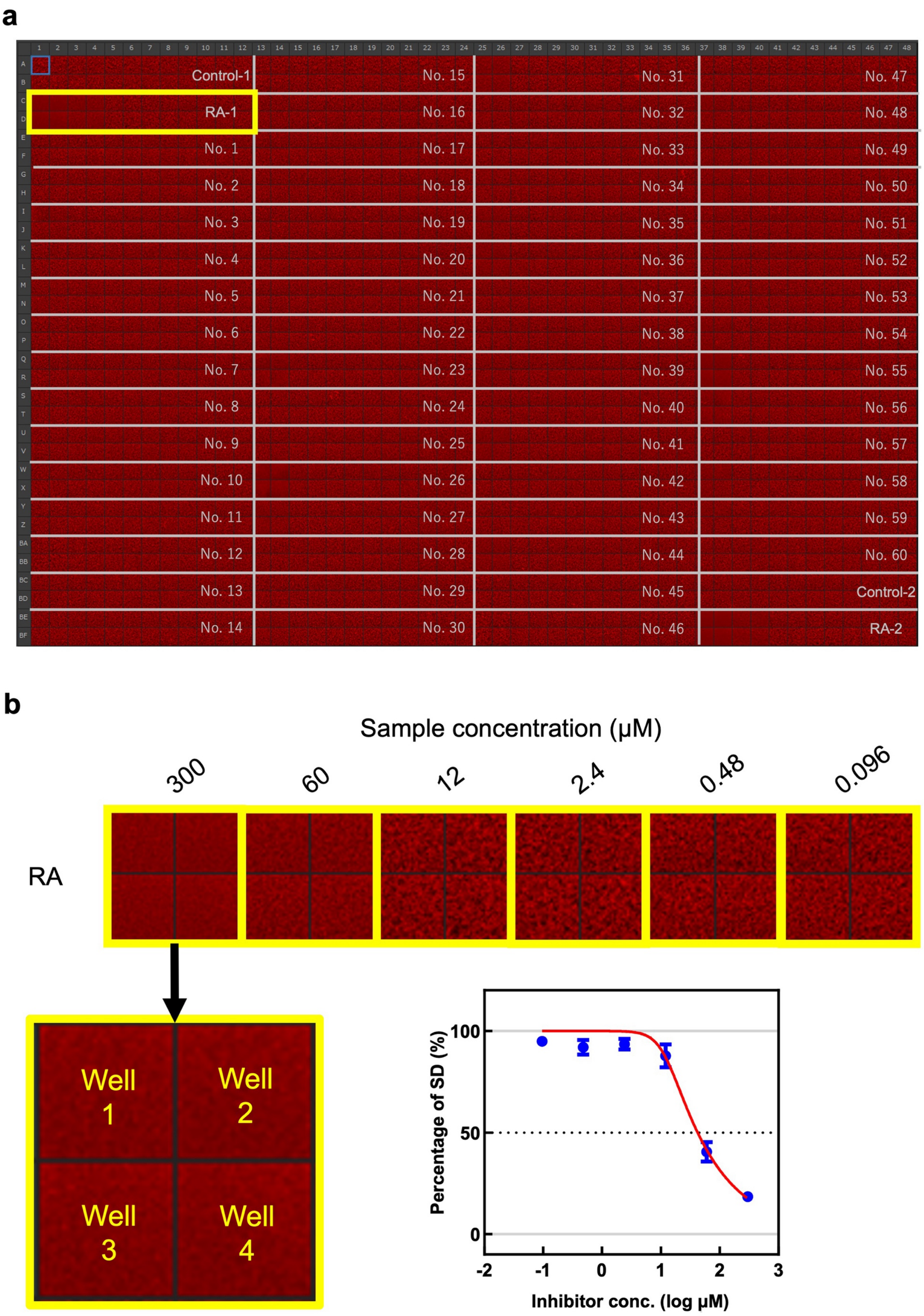

Supplementary Fig. 10. Sample distribution in a 1536-well plate. a, Distribution of each sample in a 1536-well plate. A single 1536-well plate can accommodate the analysis of 64 samples

(n = 4). The yellow-boxed region shows the concentration series of rosmarinic acid (RA) as a positive control experiment. **b**, Enlarged image of yellow-boxed region in panel a. RA was diluted 5-times by a 5-fold dilution series, and injected into a 1536-well plate in the order of well-1 to well-4. 25 μM Aβ and 25 nM QDAβ were incubated with 300, 60, 12, 2.4, 0.48, or 0.096 μM RA in a 1536-well plate for 24 h at 37°C, and the center of each well was observed by fluorescence microscopy. The EC_{50} value of RA in NbM Sup Alb(-) was estimated using Prism software (GraphPad Software, Boston, MA, USA), as described in the Methods section. Error bars represent ± SDs of the mean values from fluorescence intensities (n = 4 separate experiments).

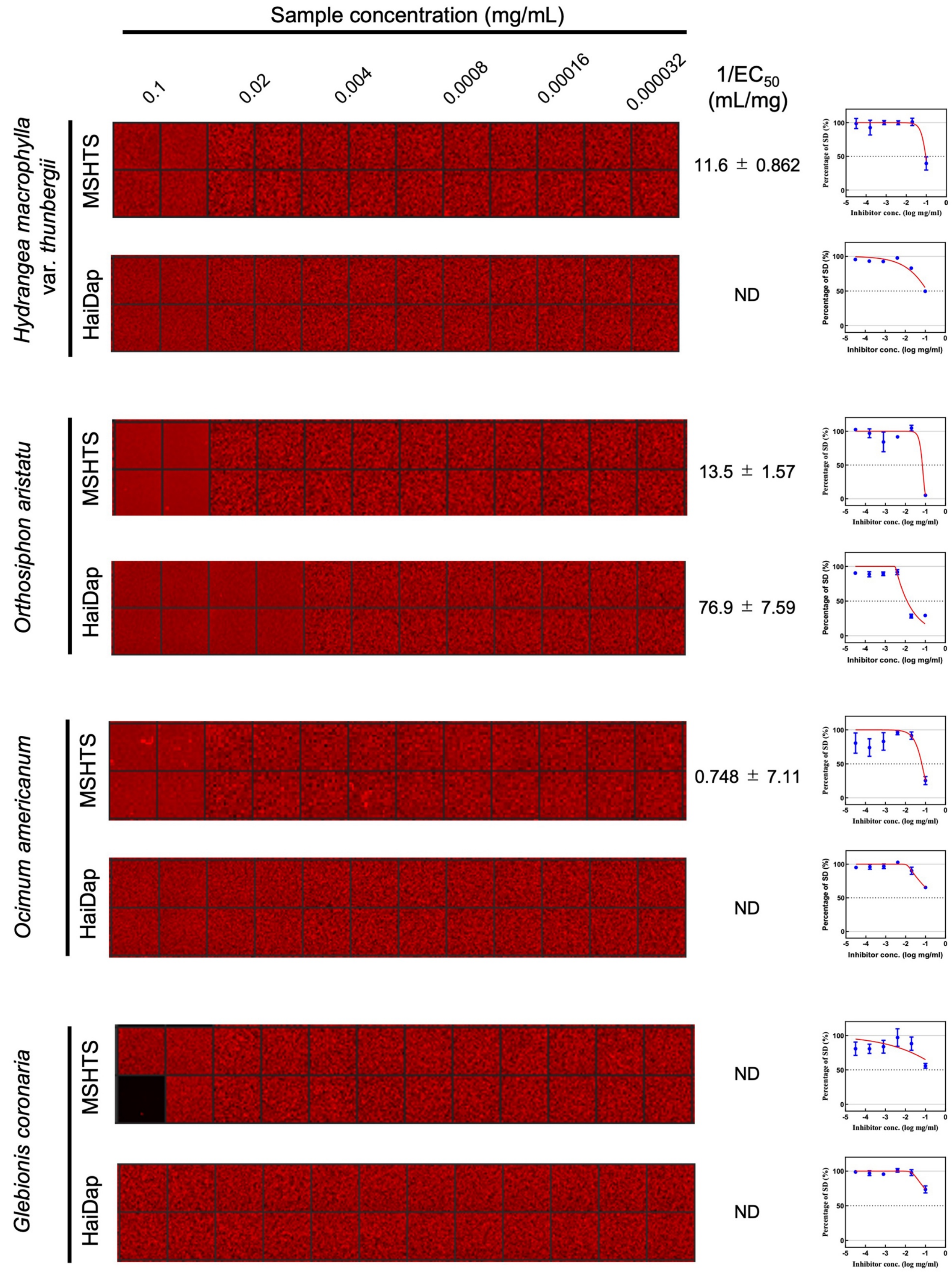
Sample concentration (mg/mL)
0.1
0.02
0.004
0.0008
0.00016
0.000032
1/EC50 (mL/mg)
Hydrangea macrophylla var. thunbergii
MSHTS
11.6 ± 0.862
HaiDap
ND
Orthosiphon aristatu
MSHTS
13.5 ± 1.57
HaiDap
76.9 ± 7.59
Ocimum americanum
MSHTS
0.748 ± 7.11
HaiDap
ND
Glebionis coronaria
MSHTS
ND
HaiDap
ND
Percentage of SD (%)
Inhibitor conc. (log mg/ml)

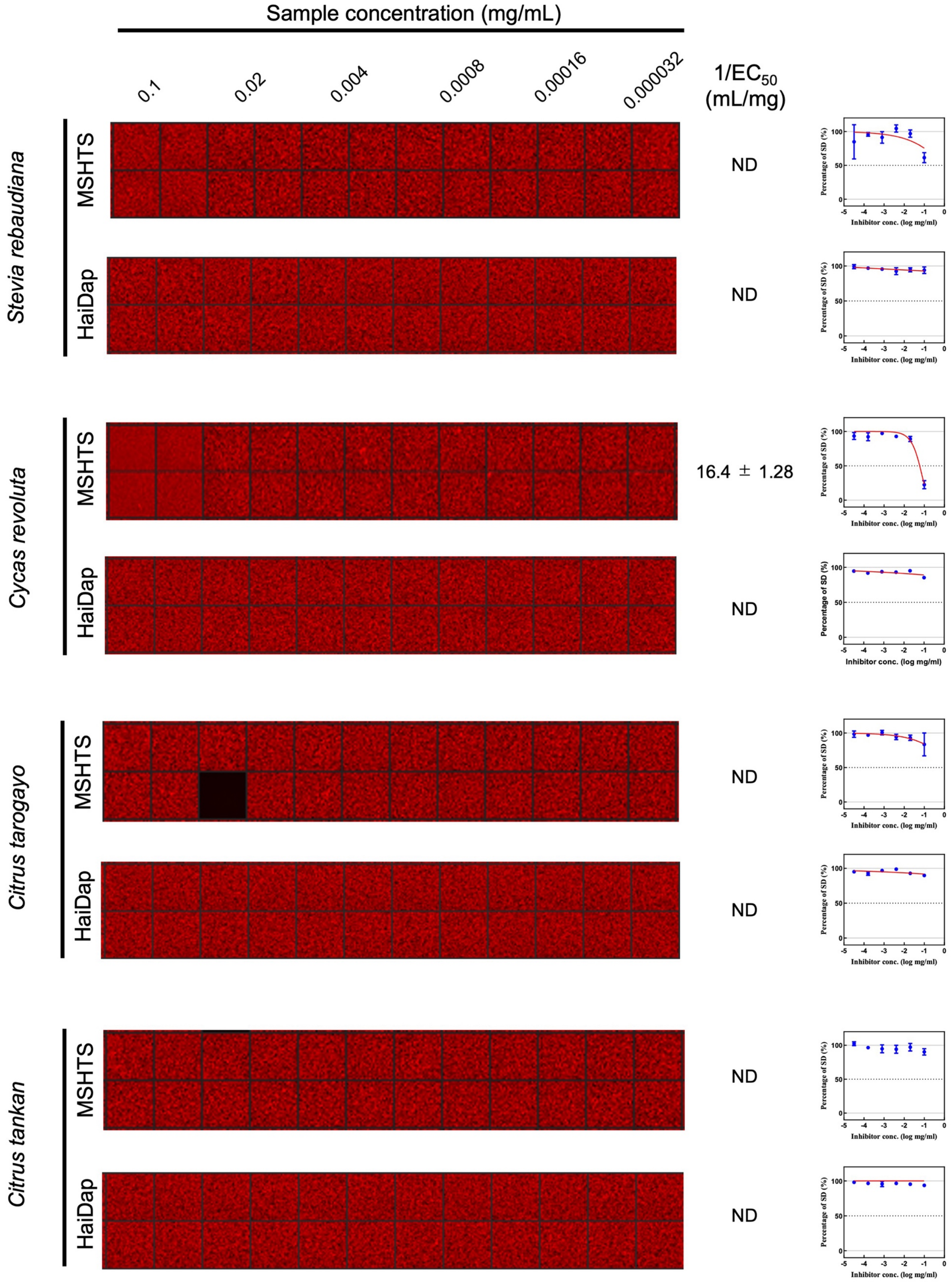

Sample concentration (mg/mL)
0.1
0.02
0.004
0.0008
0.00016
0.000032
1/EC50 (mL/mg)
Stevia rebaudiana
MSHTS
ND
HaiDap
ND
Cycas revoluta
MSHTS
16.4 ± 1.28
HaiDap
ND
Citrus tarogayo
MSHTS
ND
HaiDap
ND
Citrus tankan
MSHTS
ND
HaiDap
ND
Percentage of SD (%)
Inhibitor conc. (log mg/ml)

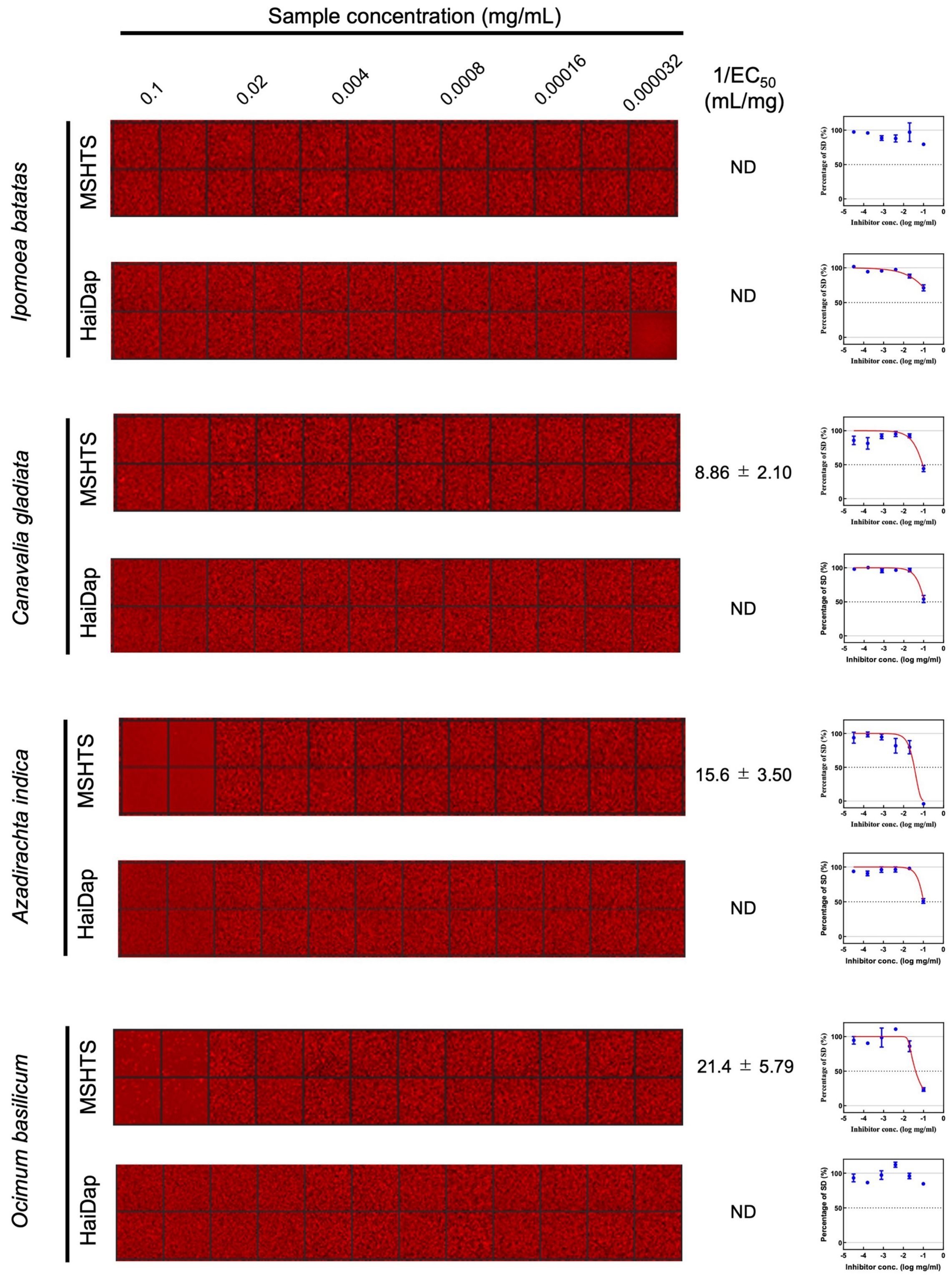

Sample concentration (mg/mL)
0.1
0.02
0.004
0.0008
0.00016
0.000032
1/EC50 (mL/mg)
Ipomoea batatas
MSHTS
ND
HaiDap
ND
Canavalia gladiata
MSHTS
8.86 ± 2.10
HaiDap
ND
Azadirachta indica
MSHTS
15.6 ± 3.50
HaiDap
ND
Ocimum basilicum
MSHTS
21.4 ± 5.79
HaiDap
ND
Percentage of SD (%)
Inhibitor conc. (log mg/ml)

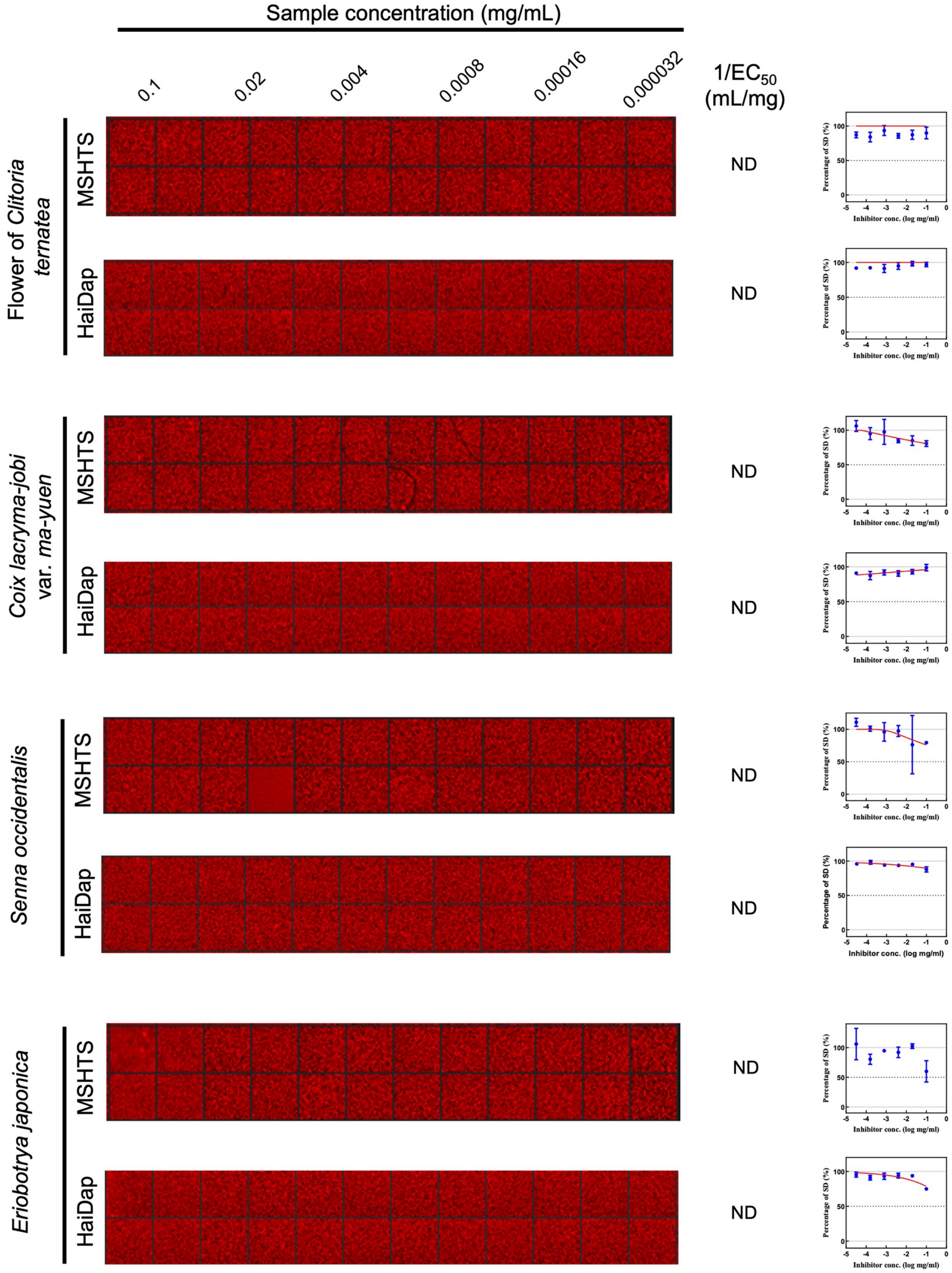
Sample concentration (mg/mL)
0.1
0.02
0.004
0.0008
0.00016
0.000032
1/EC50 (mL/mg)
Flower of Clitoria ternatea
MSHTS
ND
HaiDap
ND
Coix lacryma-jobi var. ma-yuen
MSHTS
ND
HaiDap
ND
Senna occidentalis
MSHTS
ND
HaiDap
ND
Eriobotrya japonica
MSHTS
ND
HaiDap
ND
Percentage of SD (%)
Inhibitor conc. (log mg/ml)

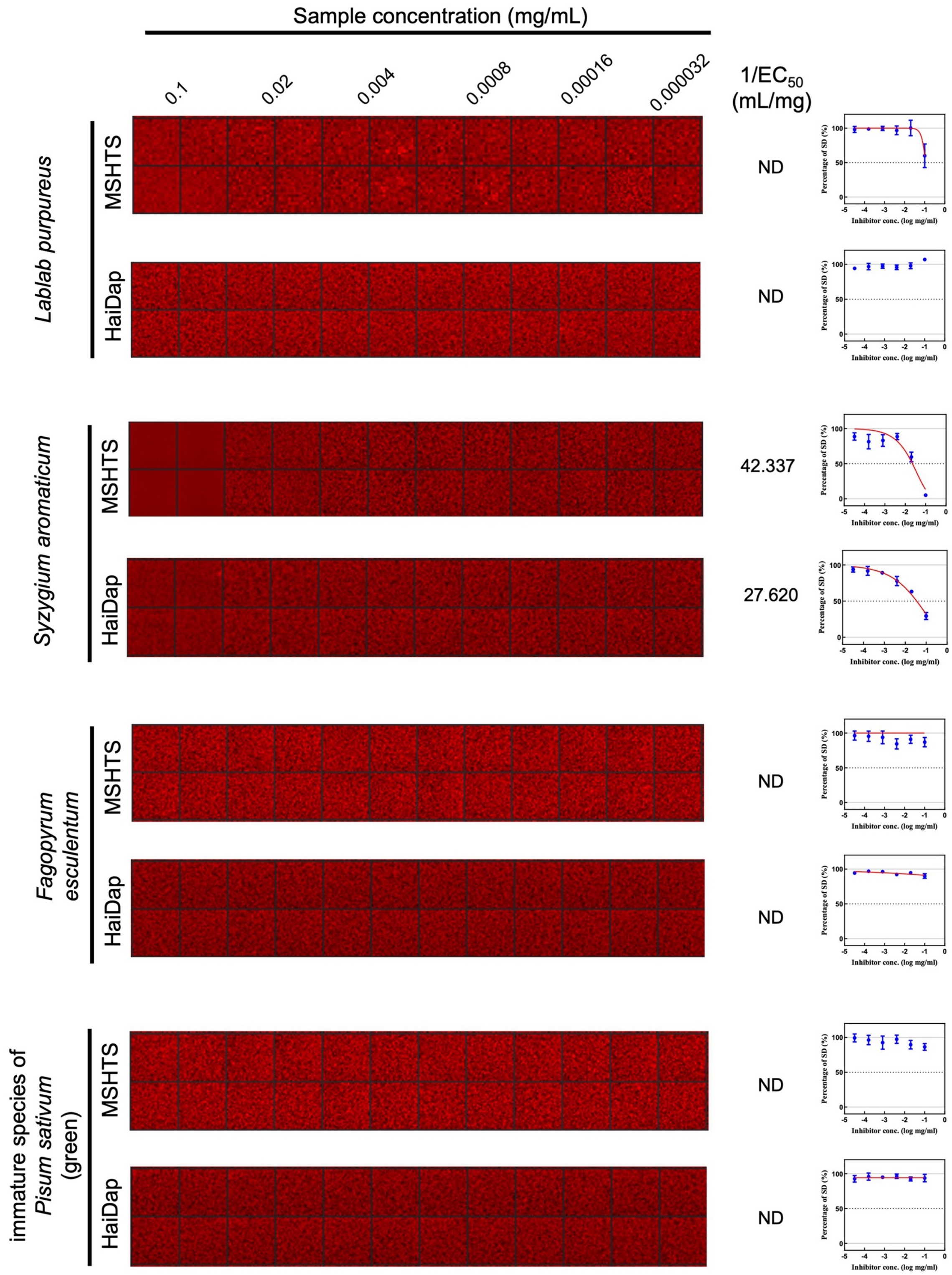
Sample concentration (mg/mL)
0.1
0.02
0.004
0.0008
0.00016
0.000032
1/EC50 (mL/mg)
Lablab purpureus
MSHTS
ND
HaiDap
ND
Syzygium aromaticum
MSHTS
42.337
HaiDap
27.620
Fagopyrum esculentum
MSHTS
ND
HaiDap
ND
immature species of Pisum sativum (green)
MSHTS
ND
HaiDap
ND
Percentage of SD (%)
Inhibitor conc. (log mg/ml)

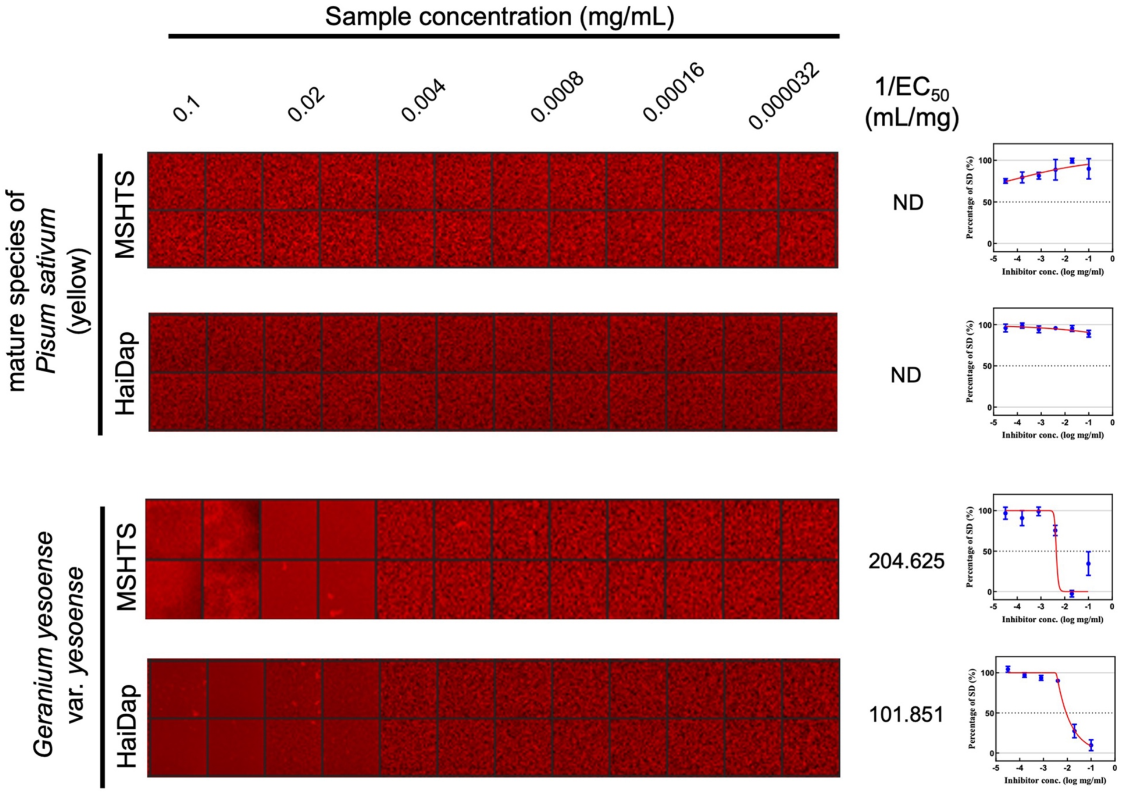

Supplementary Fig. 11. Evaluation of Aβ aggregation inhibitory activity of 22 plant extracts using MSHTS and HaiDap systems. The dilution of extract samples was repeated five times, so that six dilution series were prepared for each sample (0.1, 0.02, 0.004, 0.0008, 0.00016, and 0.000032 mg/mL). The EC_{50} values of 22 plant extracts were determined by an automated HaiDap system. The black wells are wells into which samples could not be injected due to a malfunction in the tip attachment of the automated workstation. Error bars represent ± SDs of the mean values from fluorescence intensities (n = 4 separate experiments).

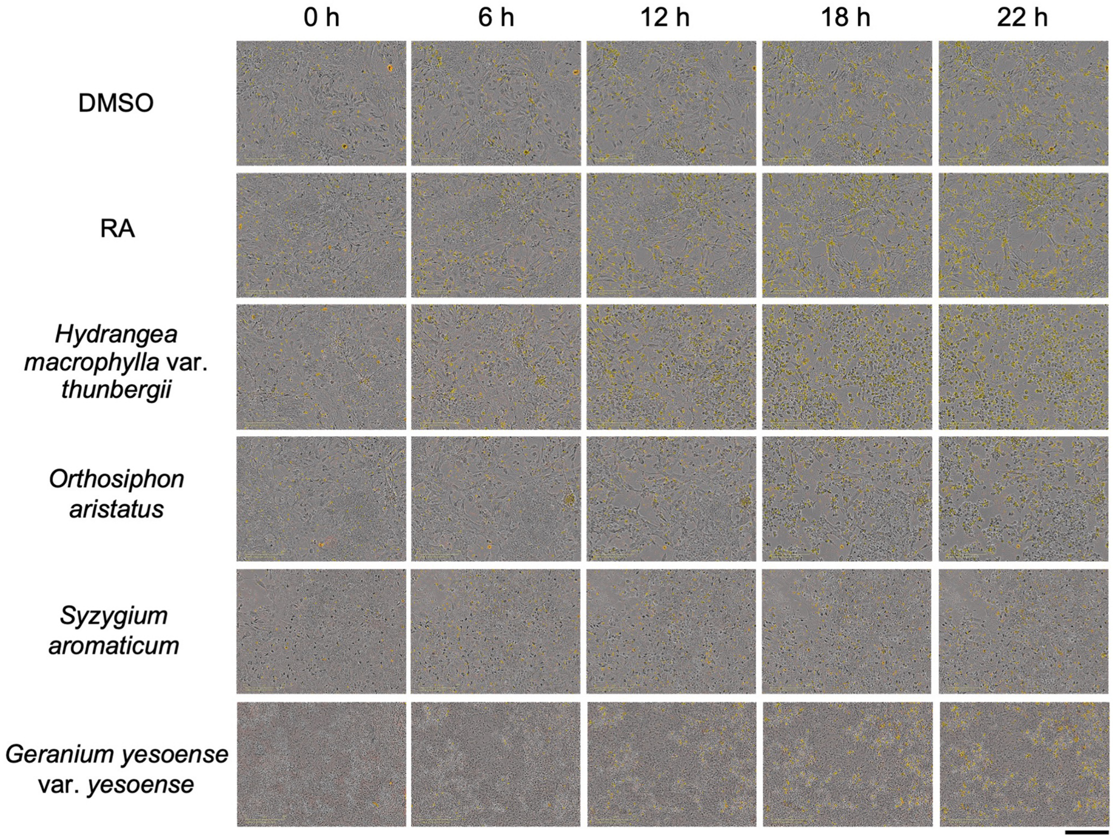

Supplementary Fig. 12. The Aβ aggregation process on neuron-differentiated iPSCs in indicated conditions and times was measured using the Incucyte Live Imaging System. To measure levels of aggregated Aβ, the neurons were cultured in NbM containing an extract of *Hydrangea macrophylla* var. *thunbergia*, *Orthosiphon aristatus*, and *Syzygium aromaticum*, and *Geranium yesoense* var. *yesoense*. DMSO is a control experiment that did not contain any plant extract, and RA is an experiment in which rosmarinic acid was added instead of the extract. Bar = 200 µm. Standby time to maintain temperature constant inside the cell culture plate was as long as 2 h from the start of these assays. Yellow indicates Aβ aggregation.

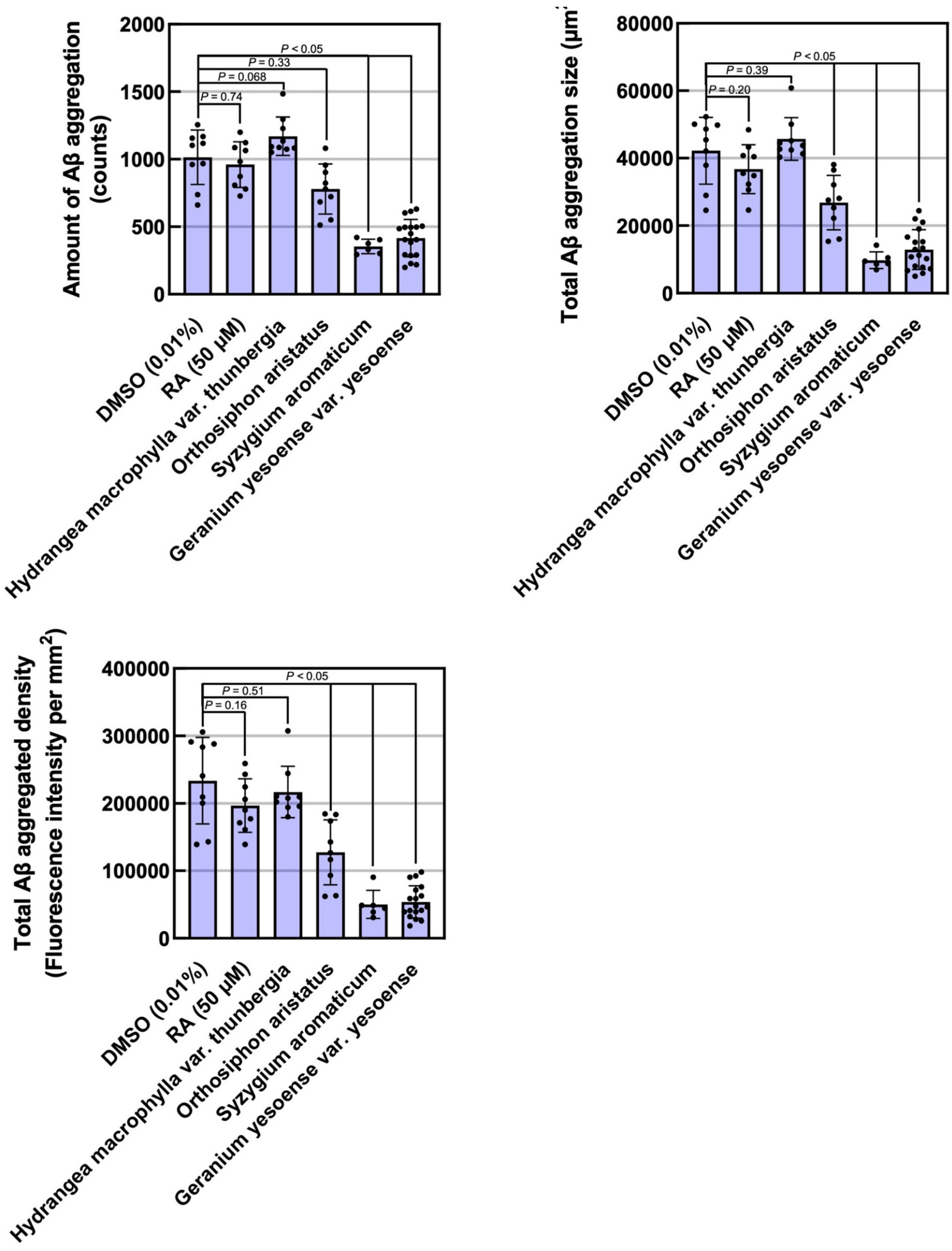

Supplementary Fig. 13. Quantification of Aβ aggregation on the cell surface in indicated conditions and times measured using the Incucyte Live Imaging System. To measure levels of aggregated Aβ, neurons were cultured in Neurobasal® Medium containing an extract of *Hydrangea macrophylla* var. *thunbergia*, *Orthosiphon aristatus*, *Syzygium aromaticum*, and *Geranium yesoense* var. *yesoense*. Quantitative data after 22 h of incubation extracted from the results in Fig. 7**d**. Error bars represent ± SEs of the mean values. $p < 0.05$, Student's t-test

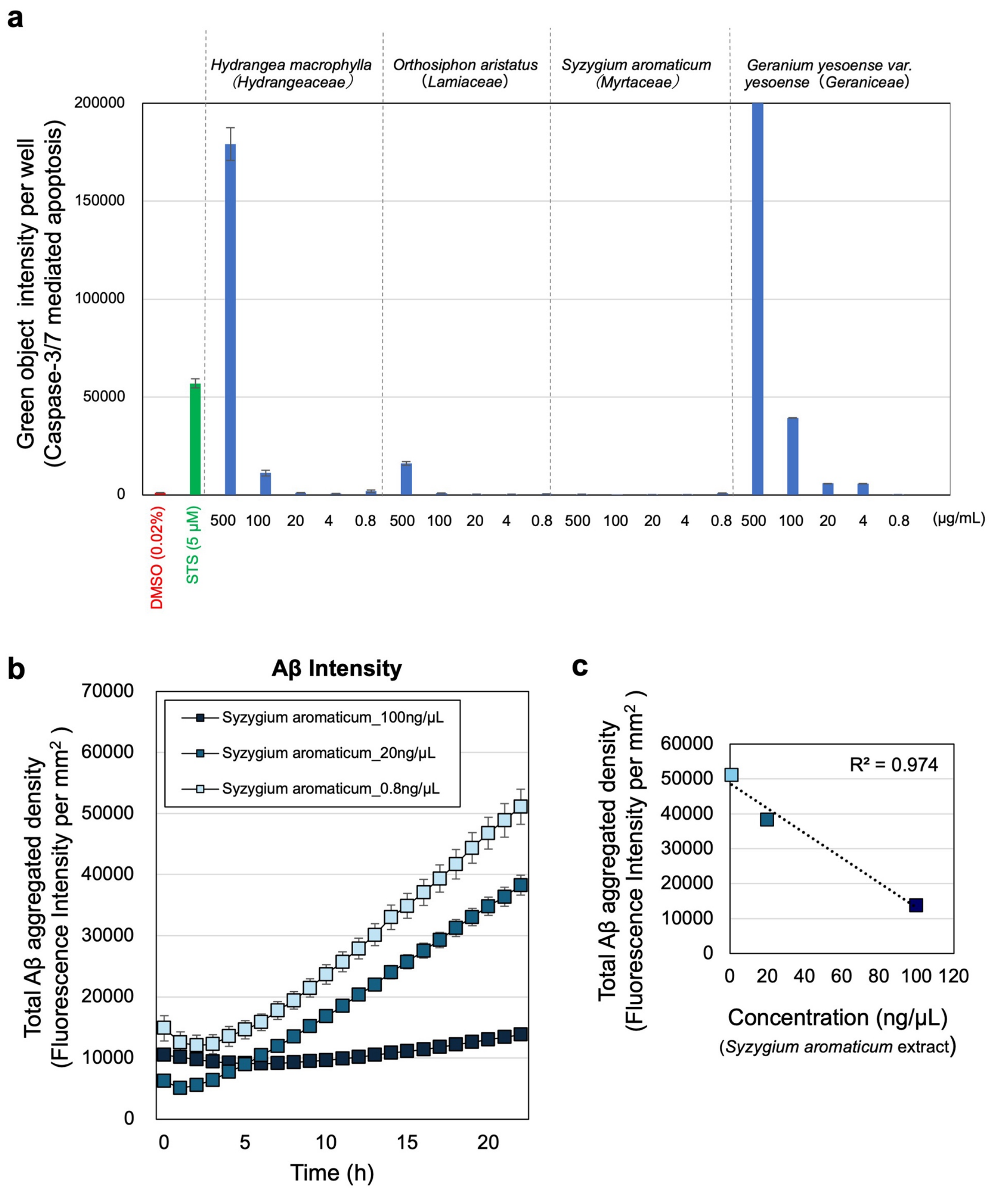

Supplementary Fig. 14. Dose-dependent evaluation of the *Syzygium aromaticum* extract concentration and the inhibition of Aβ aggregation. a, Cytotoxicity assessment. To evaluate whether the extracts of four representative plants (*H. macrophylla*, *O. aristatus*, *S. aromaticum*, and *G. yesoense* var. *yesoense*), selected by the HaiDap method, could induce caspase 3/7-mediated apoptosis in neurons in a concentration-dependent manner by using Incucyte. Evaluation of Caspase 3/7-mediated induction of cell death in neurons by four plant extracts (medium containing 0.8-500 µg/mL *H. macrophylla* extract, medium containing 0.8-500 µg/mL

O. aristatus extract, medium containing 0.8-500 µg/mL *S. aromaticum* extract, medium containing 0.8-500 µg/mL *G. yesoense* var. *yesoense* extract). Error bar represents of the mean values from ± SEs of the mean values (for each sample: n = 18). **b,** iPSC-based screening. To assess whether the presence of different concentrations of the *S. aromaticum* extract—0.8, 20, or 100 ng/µL—in medium would lead to an alteration in the amount of Aβ aggregates that accumulated in neurons (for each sample: n = 56). The negative control without *S. aromaticum* extract (0 ng/µL) corresponds to the DMSO control in Fig. 7d and is therefore not shown here. **c**, The inhibitory effect of a high-concentration of *S. aromaticum* extract on Aβ aggregation is robust, and a correlation exists between concentration and the inhibitory effect.

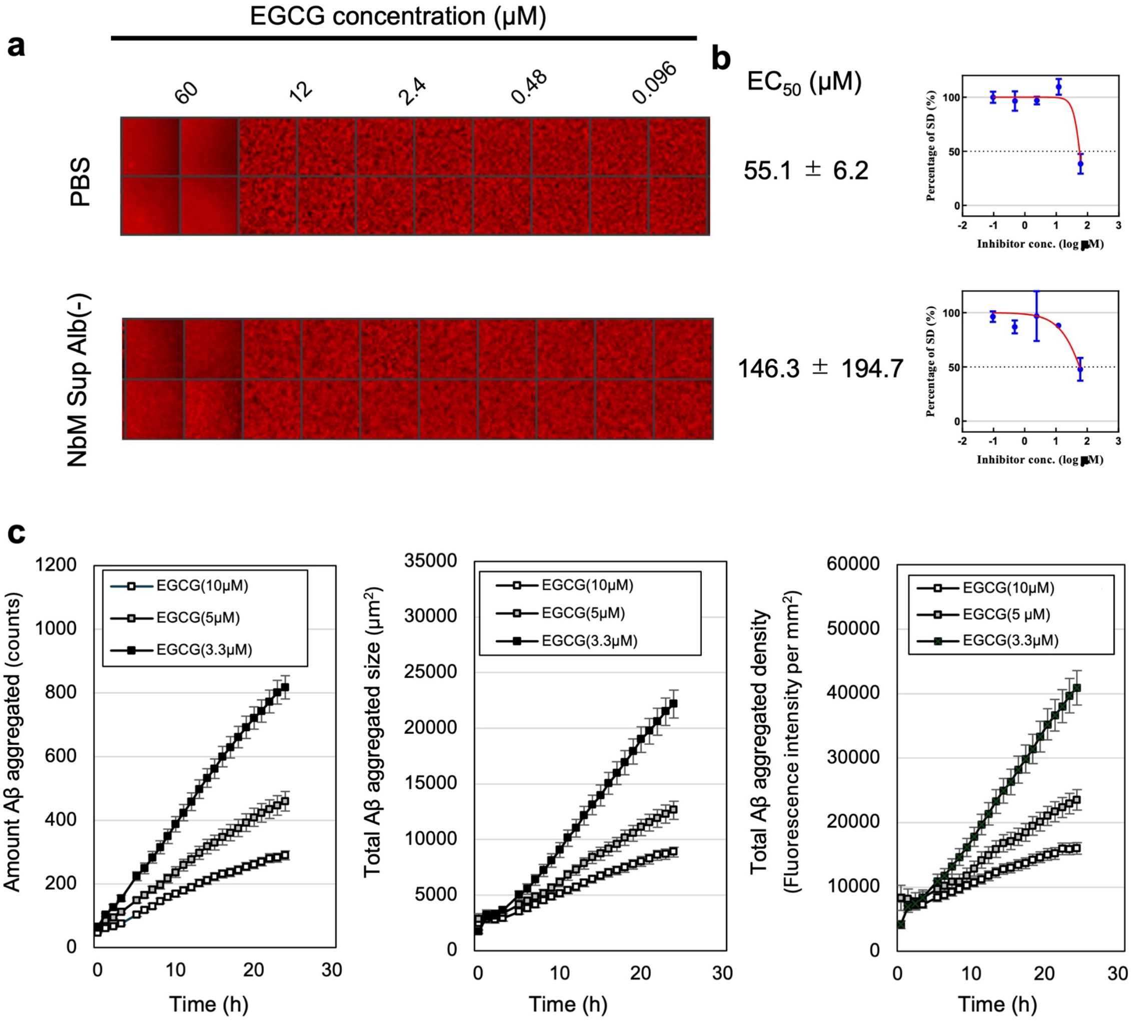

Supplementary Fig. 15. The evaluation of Aβ aggregation inhibitory activity of EGCG. a, The evaluation of EGCG using MSHTS system and HaiDap system. 25 nM QDAβ_{40} and 25 μM Aβ_{42} were incubated with five concentrations of EGCG at 37°C for 24 h. **b**, Estimations of EC_{50} of EGCG. Error bars represent ± SDs of the mean values from fluorescence intensities (n = 4 separate experiments). **c**, The evaluation of dose-dependent inhibition effects of EGCG using iPSC-based screening. Aβ counts, Aβ aggregation size and total red object integrated intensity, expressed as red calibrated units × μm^2/well, were used in the QDAβ method. Error bars represent ± SEs of the mean values from 9 independent regions. The negative control without EGCG (0 μM) corresponds to the DMSO control in Fig. 7d.

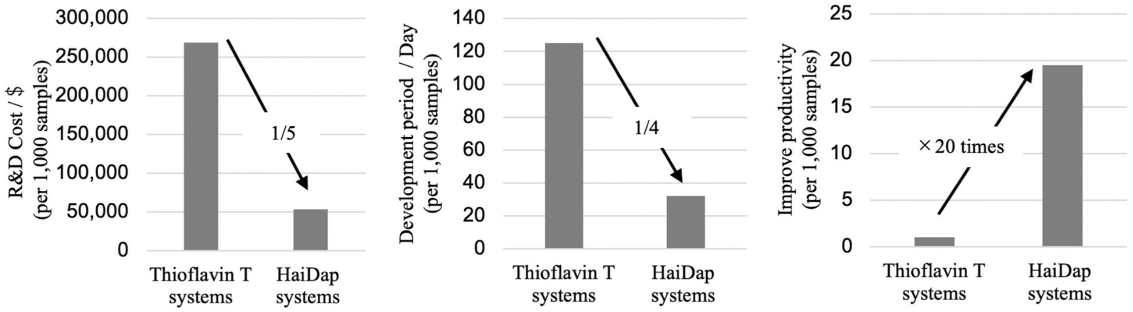

Sample	Screening model	Cost / $	Numbers/Sample	Dose-response study	Throughput (Sample/Run)	One Run (Day)	Plate Type	Analysis period (Day)	CV Value (Control Sample)
1000	Thioflavin T system	268,500	Quadruplecation	6	4	0.5	96	125	About 15 % <
1000	HaiDap system	53,700	Quadruplecation	6	64	2	1536	32	About 15 % <

Improve productivity (at least)	Spectral Selectivity
1	Low
19.5	High

Supplementary Fig. 16. The HaiDap system can reduce R&D cost by about one-fifth compared to the ThT system. The HaiDap system was compared with a well-known method for assessing the inhibition of Aβ aggregation, the ThT system using a standard 96-well plate. Estimating the cost of 1000 samples tested under the same conditions (n = 4, six dose-dependent), HaiDap can reduce R&D cost by about one-fifth compared to the ThT system and the development period can be shortened by one quarter. Calculating productivity from the perspective of R&D cost and period, it can be inferred that HaiDap is a system with at least 20-fold greater productivity than the ThT system. In recent years, ThT kits using 384-well plates have become commercially available, although the HaiDap system has significant advantages.

Supplementary table

Plant extract		MSHTS	HaiDap
Sample Name	Site	1/EC_{50} average	1/EC_{50} averege
Hydrangea macrophylla var. *thunbergii*	Leaf/Stem	11.572	N.D
Orthosiphon aristatu	Leaf/Stem	13.507	76.938
Ocimum americanum	Leaf/Stem	0.748	N.D
Glebionis coronaria	Leaf/Stem	N.D	N.D
Stevia rebaudiana	Leaf/Stem	N.D	N.D
Cycas revoluta	Seed	16.407	N.D
Citrus tarogayo	Fruit	N.D	N.D
Citrus tankan	Fruit	N.D	N.D
Ipomoea batatas	Root	N.D	N.D
Canavalia gladiata	Seed	8.861	N.D
Azadirachta indica	Leaf/Stem	15.615	N.D
Ocimum basilicum	Leaf/Stem	21.444	N.D
Flower of Clitoria ternatea	Flower	N.D	N.D
Coix lacryma-jobi var. *ma-yuen*	Seed	N.D	N.D
Senna occidentalis	Seed	N.D	N.D
Eriobotrya japonica	Leaf/Stem	N.D	N.D
Lablab purpureus	Leaf/Stem	N.D	N.D
Syzygium aromaticum	Flower buds	42.337	27.620
Fagopyrum esculentum	Seed	N.D	N.D
Immature *species of Pisum sativum* (green)	Seed	N.D	N.D
Mature *species of Pisum sativum* (yellow)	Seed	N.D	N.D
Geranium yesoense var. *yesoense*	Leaf/Stem	204.625	101.851

Supplementary Table 1. Aβ aggregation inhibitor activity (1/EC_{50}) of plant extracts calculated by the MSHTS and HaiDap systems. The MSHTS system tended to detect samples more easily than the HaiDap system. Therefore, HaiDap may be a system that makes it easier to narrow down the identification of extracts or compounds.
